# Disrupted Balance of Long- and Short-Range Functional Connectivity Density in Type 2 Diabetes Mellitus: A Resting-State fMRI Study

**DOI:** 10.3389/fnins.2018.00875

**Published:** 2018-11-27

**Authors:** Daihong Liu, Lihua Chen, Shanshan Duan, Xuntao Yin, Wu Yang, Yanshu Shi, Jiuquan Zhang, Jian Wang

**Affiliations:** ^1^Department of Radiology, Southwest Hospital, Third Military Medical University (Army Medical University), Chongqing, China; ^2^Department of Imaging Diagnosis, Lanzhou General Hospital of Chinese PLA Lanzhou Command (PLA No. 940 Hospital), Lanzhou, China; ^3^Department of Radiology, PLA No. 904 Hospital, Wuxi, China; ^4^Department of Endocrinology, Southwest Hospital, Third Military Medical University (Army Medical University), Chongqing, China; ^5^Medical Company, The Chinese People’s Liberation Army No.31610 Troop, Zhoushan, China; ^6^Department of Radiology, Chongqing University Cancer Hospital & Chongqing Cancer Institute & Chongqing Cancer Hospital, Chongqing, China; ^7^Key Laboratory for Biorheological Science and Technology of Ministry of Education (Chongqing University), Chongqing University Cancer Hospital & Chongqing Cancer Institute & Chongqing Cancer Hospital, Chongqing, China

**Keywords:** type 2 diabetes mellitus, cognitive impairment, resting-state functional MRI, functional connectivity density, functional connectivity

## Abstract

Previous studies have shown that type 2 diabetes mellitus (T2DM) can accelerate the rate of cognitive decline in patients. As an organ with high energy consumption, the brain network balances between lower energy consumption and higher information transmission efficiency. However, T2DM may modify the proportion of short- and long-range connections to adapt to the inadequate energy supply and to respond to various cognitive tasks under the energy pressure caused by homeostasis alterations in brain glucose metabolism. On the basis of the above theories, this study determined the abnormal functional connections of the brain in 32 T2DM patients compared with 32 healthy control (HC) subjects using long- and short-range functional connectivity density (FCD) analyses with resting-state fMRI data. The cognitive function level in these patients was also evaluated by neuropsychological tests. Moreover, the characteristics of abnormal FCD and their relationships with cognitive impairment were investigated in T2DM patients. Compared with the HC group, T2DM patients exhibited decreased long-range FCD in the left calcarine and left lingual gyrus and increased short-range FCD in the right angular gyrus and medial part of the left superior frontal gyrus (*p* < 0.05, Gaussian random-field theory corrected). In T2DM patients, the FCD z scores of the medial part of the left superior frontal gyrus were negatively correlated with the time cost in part B of the Trail Making Test (ρ = -0.422, *p* = 0.018). In addition, the FCD z scores of the right angular gyrus were negatively correlated with the long-term delayed recall scores of the Auditory Verbal Learning Test (ρ = -0.356, *p* = 0.049) and the forward scores of the Digital Span Test (ρ = -0.373, *p* = 0.039). T2DM patients exhibited aberrant long-range and short-range FCD patterns, which may suggest brain network reorganization at the expense of losing the integration of long-range FCD to adapt to the deficiency in energy supply. These changes may be associated with cognitive decline in T2DM patients.

## Introduction

Type 2 diabetes mellitus (T2DM) is characterized by disordered glucose metabolism and the number of affected individuals increased rapidly from 415 million in 2015 to 425 million in 2017 according to the 8th edition of Diabetes Atlas published by the International Diabetes Federation. A growing number of publications have demonstrated that T2DM accelerates the speed of cognitive decline which could be up to twice as fast as normal aging (Koekkoek et al., 2015). However, the brain dysfunction and cognitive impairment associated with T2DM have not been fully investigated. As the brain has high energy consumption, it is vulnerable to the fluctuations in plasma glucose levels caused by T2DM. Therefore, a better understanding of the characteristics of brain dysfunction on the background of impaired energy homeostasis may enable early diagnosis and treatment.

Type 2 diabetes mellitus is associated with reduced glucose metabolism in the brain, which may result in putative reorganization of long- and short-range functional connections. The development of normal brain functional networks is characterized by a “local to distant” organization (Fair et al., 2009). Brain regions with short-range functional connections are often specialized for modular information processing and operate with lower time- and energy-cost. By contrast, long-range functional connections allow integrative information processing across distributed brain systems with higher time- and energy-cost (Sepulcre et al., 2010). The balance of long- and short-range functional connections is critical for the efficiency of cortical information communication and energy-cost (Sepulcre et al., 2010). However, T2DM patients are reported to have reduced brain glucose metabolism which is correlated with poor performance on executive and memory function tests (Garcia-Casares et al., 2014). Thus, impaired glucose homeostasis may disrupt the established balance of long- and short-range functional connections for the economical trade-off between cost and efficiency in T2DM patients (Bullmore and Sporns, 2012). As the alterations in glucose metabolism are coupled with alterations in blood-oxygen level-dependent signals, functional magnetic resonance imaging (fMRI) is advantageous in mapping the reorganization of long- and short-range functional connections (Sepulcre et al., 2010; Magistretti and Allaman, 2015).

Resting-state fMRI has been widely used to determine the aberrations in brain function in T2DM patients. On the one hand, T2DM patients exhibited altered amplitude of low frequency fluctuation in the middle temporal gyrus, lingual gyrus, postcentral gyrus and occipital lobe in functional segregation studies (Xia et al., 2013; Cui et al., 2014). On the other hand, T2DM patients showed aberrant local synchronization in the lingual gyrus, fusiform gyrus, and frontal lobe (Cui et al., 2014; Liu et al., 2016) and disrupted functional connectivity anchoring in the posterior cingulate cortex (Chen et al., 2014) or within the default mode network, frontal parietal network and sensorimotor network (Chen et al., 2015) in functional integration studies. As an approach for functional integration, the analysis of long- and short-range functional connections facilitates the investigation of brain dysfunction in patients with schizophrenia (Guo et al., 2015), minimal hepatic encephalopathy (Qi et al., 2015), non-alcoholic cirrhosis after liver transplantation (Zhang et al., 2015a), end-stage renal disease (Zhang et al., 2015b) and conduct disorder (Lu et al., 2017). Previous studies have also suggested that T2DM patients may suffer from cognition decline linked to alterations in long- and short-range brain functional synchronization and functional connectivity strength (Liu et al., 2016, 2017). In addition to the aforementioned neuroimaging indicators, the functional connectivity density (FCD), which quantifies the number of functional connections between a given voxel and the remaining voxels in the entire brain, is a graph theoretical indicator to analyze the complex brain networks (Tomasi et al., 2016). However, a map of the long- and short-range functional connections assessed using FCD remains to be delineated in T2DM patients. Furthermore, the similar definitions of FCD and degree of centrality suggest that the brain regions with aberrant FCD may play pivotal roles in global information communication (Zuo et al., 2012). Therefore, they can be considered as seed regions to calculate their functional connectivity with the whole brain as in a previous study (Cui et al., 2016) and the pattern of the changed FCD can be characterized.

In the present study, we assume that the cognitive impairment in T2DM patients may be associated with disruption of the balance between long- and short-range FCD. We first investigated the changed long- and short-range FCD pattern in T2DM patients, and then calculated the functional connectivity of the identified brain regions with the whole brain. Finally, we examined the relationships between these neuroimaging changes and cognition decline. This study may contribute to understanding the reorganization of brain functional architecture accompanying cognitive decline in T2DM patients.

## Materials and Methods

### Subjects

This study recruited 32 T2DM patients from inpatients and communities and 32 healthy controls (HC) from communities during December 2013 and November 2016. The T2DM group and HC group were matched for age, sex, education, and body mass index (BMI). T2DM diagnosis conformed to the criteria published by the World Health Organization in 1999 (Alberti and Zimmet, 1998). Subjects in the two groups were included according to the following criteria: (1) 45 years ≤ age ≤ 70 years; (2) education ≥6 years; (3) right-handedness; (4) normal general cognitive level defined by a Mini-Mental State Examination (MMSE) score ≥25; (5) T2DM duration of patients at least 1 year. Key exclusion criteria for both groups were as follows: (1) brain structural abnormalities including trauma, stroke, tumor, or white matter changes with a rating score ≥2 (Wahlund et al., 2001); (2) neurological or psychiatric diseases including major depression, dementia, schizophrenia and epilepsy; (3) pregnancy, extremity disability, and the loss of audition or vision; (4) contraindications for MRI examination. T2DM patients with diabetic foot, retinopathy, nephropathy and other complications were also excluded. The Medical Research Ethics Committee of the Southwest Hospital (Chongqing, China) approved the study protocol in accordance with the recommendations of the declaration of Helsinki for investigation of human participants. All participants provided written informed consent after being informed of the study details.

### Clinical Evaluation

All subjects underwent clinical evaluation with a standardized protocol. Demographic information included age, sex and education. The physical data included handedness, height, weight, and resting arm arterial blood pressure. BMI was calculated according to height and weight [(weight in kg)/(height in m)^2^]. Medical history and current medications were also recorded. The dates of T2DM diagnoses were recorded to compute the disease duration. After an overnight abrosia, venous blood samples were collected by venipuncture for the evaluation of biometric measurements, including glucose parameters, lipid parameters, renal function parameters, thyroid function parameters, and homocysteine (listed in Table 1). Fasting insulin and plasma glucose were used to calculate the updated homeostasis model assessment of insulin resistance (HOMA2-IR) index with HOMA2 Calculator v2.2.3 software^1^.

**Table 1:** Demographic and clinical data of all included subjects.

	T2DM	HC	*p*-value
Age (years)	58.09 ± 7.26	56.88 ± 5.01	0.437
Sex (male:female)	19:13	18:14	0.800^a^
Education (years)	9.00 (9.00, 12.00)	12.00 (9.00, 12.00)	0.122^b^
T2DM duration (years)	10.00 (4.00, 12.50)	–	–
BMI (kg/m^2^)	24.40 ± 2.73	23.89 ± 4.40	0.585
Systolic blood pressure (mmHg)	131.91 ± 17.23	133.72 ± 17.43	0.677
Diastolic blood pressure (mmHg)	82.00 ± 8.99	79.66 ± 10.06	0.330
HbA_1c_ (%)	8.30 ± 1.88	5.62 ± 0.39	<0.001
HbA_1c_ (mmol/mol)	67.25 ± 20.59	38.00 ± 4.28	<0.001
Fasting plasma glucose (mmol/L)	7.59 ± 2.82	5.25 ± 0.45	<0.001
Fasting insulin (mIU/L)	14.86 (9.66, 25.02)	12.85 (9.04, 17.35)	0.272^b^
Fasting C-peptide (ng/ml)	1.89 ± 1.08	2.34 ± 1.04	0.106
HOMA2-IR	0.29 (0.20, 0.53)	0.25 (0.17, 0.33)	0.124^b^
Total cholesterol (mmol/L)	5.01 ± 1.13	5.02 ± 0.98	0.955
Triglyceride (mmol/L)	1.64 (1.27, 3.00)	1.31 (0.89, 1.56)	0.018^b^
HDL cholesterol (mmol/L)	1.04 ± 0.23	1.39 ± 0.33	<0.001
LDL cholesterol (mmol/L)	3.21 ± 0.92	3.22 ± 0.75	0.944
Homocysteine (μmol/L)	16.01 ± 10.85	10.80 ± 4.47	0.260
Blood urea nitrogen (mmol/L)	6.08 ± 2.36	5.67 ± 1.23	0.392
Serum creatine (μmol/L)	73.38 ± 28.57	78.31 ± 16.20	0.399
Cystatin C (mg/L)	0.71 (0.63, 0.88)	0.79 (0.69, 0.86)	0.151^b^
Uric acid (μmol/L)	302.38 ± 76.77	325.88 ± 73.25	0.215
Free triiodothyronine, FT3 (pmol/L)	4.22 ± 0.86	5.05 ± 0.57	<0.001
Free thyroxine, FT4 (pmol/L)	15.09 ± 2.03	16.38 ± 2.02	0.014
Thyroid stimulating hormone, TSH (mIU/L)	1.96 ± 1.12	2.33 ± 1.42	0.251

*p < 0.05 indicates statistically significant. ^a^The Chi-square test for dichotomous data. ^b^The Mann-Whitney U-test for non-normally distributed data [median (QR)]. Two sample t-test for normally distributed continuous data (means ± SD).*

### Cognitive Assessment

Cognitive assessments were performed before MRI scanning. A battery of neuropsychological tests in a fixed order was used to assess the general cognitive level and major cognitive domains. The general cognitive level was evaluated by the MMSE and Montreal Cognitive Assessment (MoCA) tests. The executive function and psychomotor speed were evaluated by the Trail Making Test (TMT, including parts A and B) (Bowie and Harvey, 2006). Mental flexibility was evaluated by the Verbal Fluency Test (VFT) (Diamond, 2013). Working memory was evaluated with the Digital Span Test (DST, including forward and backward) (Diamond, 2013). Episodic memory was evaluated by the Auditory Verbal Learning Test (AVLT, including immediate recall, short-term delayed recall, long-term delayed recall, long-term delayed recognition and total score) (Zhao et al., 2015). In addition, depression was evaluated with the Hamilton Depression Rating Scale-24 item (HAMD) to exclude cases with major depression. The test battery was administered by a trained neuropsychologist blinded to the grouping situation. It took approximately 60 min/subject to complete all the tests.

### MRI Scan Protocol

MRI scanning was carried out with a 3.0-T MR scanner (Trio, Siemens Medical, Erlangen, Germany) using a 12-channel head coil on the same day as the clinical evaluation and cognitive assessment. Subjects were awake with their eyes closed and were relaxed during the scan. They were scanned in the supine and head-first position, with earplugs to alleviate the noise and cushions to restrict head motion. The T2-weighted images and fluid attenuated inversion recovery (FLAIR) images were acquired for radiological evaluation. The scan parameters were as follows: T1-weighted structural images were acquired using volumetric 3D magnetization prepared by rapid-acquisition gradient-echo (MP-RAGE) sequence for radiological evaluation and the anatomical segmentation and spatial normalization in preprocessing. The scan parameters were as follows: TR = 1900 ms, TE = 2.52 ms, FA = 9°, FOV = 256 × 256 mm^2^, slices = 176, thickness = 1 mm, matrix = 256 × 256 and voxel size = 1 × 1 × 1 mm^3^, sagittally scanned and lasted 4 min and 26 s. Resting-state functional images were collected using an echo planar imaging (EPI) sequence for functional processing: TR = 2000 ms, TE = 30 ms, FA = 90°, FOV = 192 × 192 mm^2^, slices = 36, thickness = 3 mm, matrix = 64 × 64 and voxel size = 3 × 3 × 3 mm^3^, 240 volumes, transversely scanned and lasted 8 min and 8 s.

### MRI Data Processing

Two radiologists with at least 5-year work experience reviewed the T1-weighted, T2-weighted and FLAIR images to identify brain structural abnormalities and to rate white matter changes. None of the subjects met the exclusion criteria. The structural and functional images underwent preprocessing with a standard protocol in Graph Theoretical Network Analysis Toolbox version 1.2.1 (GRETNA V1.2.1) (Wang et al., 2015) as follows: (1) The DICOM data were transformed into NIfTI format. (2) The first 10 volumes of individual functional images were then removed for magnetization equilibrium. (3) Next, slice timing was performed to correct the temporal offsets between slices. (4) Realignment was performed to make each part of the brain across volumes in the same position. (5) Spatial normalization was performed to warp individual functional images to standard Montreal Neurological Institute (MNI) space derived from T1 images segmentation. (6) Detrend was applied to reduce the systematic drift in the signal. (7) The data were bandpass filtered (0.01–0.08 Hz) to reduce the effects of low frequency drift and physiological noises at high-frequency band. (8) Covariate regression was applied to remove the confounding variables, including head motion profiles, the cerebrospinal fluid signal, the white matter signals and the global signal.

Voxel-based degree analysis was conducted within a gray matter mask on the basis of preprocessed images. The connectional threshold of FCD was set at 0.3 (Tomasi et al., 2016). It has been proven that 75 mm approximately reflects the true physical distance of connections between regions (He et al., 2007). Therefore, the sum of functional connectivity between a given voxel and other voxels beyond the sphere radius of 75 mm were defined as long-range FCD, whereas the sum of functional connectivity between a given voxel and other voxels within the sphere radius of 75 mm were defined as short-range FCD (Guo et al., 2015). Taking the sign into consideration, the FCD can be classified into four categories: long-range positive/negative FCD (lpFCD and lnFCD) and short-range positive/negative FCD (spFCD and snFCD). Spatial smoothing with 4 mm full-width half-maximum was used to improve the signal-to-noise ratio of FCD maps (Tomasi and Volkow, 2010). The brain regions with aberrant FCD obtained from the subsequent two-sample *t*-test were saved as seeds for the functional connectivity calculation. The functional connectivity calculation was performed with Resting-State fMRI Data Analysis Toolkit version 1.8 (REST V1.8) software on the basis of preprocessed images. To facilitate the statistical analyses, Fisher transformation (*r*-to-*z* transformation) was applied to normalize the distribution of *Pearson* correlation coefficient values of functional connectivity.

### Statistical Analyses

Numeric data analysis was conducted with SPSS software (version 20.0; IBM Corp., Armonk, NY, United States). Firstly, the *Kolmogorov-Smirnov* test was applied to confirm normal distribution of the data. According to the results, the two-sample *t*-test was applied to normally distributed continuous data, whereas the *Mann-Whitney U*-test was applied to non-normally distributed data comparisons between the T2DM group and HC group. The inter-group comparison of dichotomous data (sex) was performed using the *Chi*-*square* test. Values of *p* < 0.05 were considered statistically significant.

Functional connectivity density and functional connectivity maps analyses were conducted with the Statistical Analysis module of Data Processing & Analysis of Brain Imaging version 2.3 (DPABI V2.3). Firstly, the one-sample *t*-test was performed to confirm the FCD and functional connectivity distribution pattern with the base of “0” in each group. The two-sample *t*-test was then performed to compare the differences in FCD between the T2DM group and HC group, with age, sex, education, BMI, Power framewise displacement for head motion (Power et al., 2013) and the blood biometric measurements that showed significant differences (with the exception of glycemic measurements) entered as covariates. The resulting maps were multiple comparisons corrected with the Gaussian random-field theory (voxel *p* = 0.01, cluster *p* < 0.05). Z scores of T2DM patients were extracted from significantly changed brain regions according to the inter-group FCD comparison. Finally, *Pearson* correlation analyses were conducted to investigate the relationships among the changed FCD, neuropsychological test scores and clinical data after adjustment for age, sex, education, BMI and the blood biometric measurements that showed significant differences (with the exception of glycemic measurements) using SPSS software.

## Results

### Demographic and Clinical Data Comparisons

The T2DM patients were not significantly different to the HC group in terms of age, sex, education, BMI, blood pressure, fasting insulin, fasting C-peptide, HOMA2-IR, total cholesterol, low-density lipoprotein (LDL) cholesterol, homocysteine, blood urea nitrogen, serum creatine, cystatin C, uric acid, and thyroid stimulating hormone (*p* > 0.05). As expected, the levels of HbA_1c_ and fasting plasma glucose were elevated in T2DM patients. In addition, higher triglyceride and lower high-density lipoprotein (HDL) cholesterol, free triiodothyronine (FT3) and free thyroxine (FT4) levels were observed in T2DM patients (*p* < 0.05, Table 1).

### Neuropsychological Tests Comparisons

The T2DM patients scored lower in the MMSE, MoCA, DST forward, and AVLT (including short-term delayed recall, long-term delayed recall, long-term delayed recognition and total score) tests, and took longer to finish the TMT-B test (*p* < 0.05). There were no significant inter-group differences in the other neuropsychological tests (Table 2).

**Table 2:** Comparisons of neuropsychological test performance between the T2DM group and HC group.

	T2DM	HC	*p-*value
**General cognition**			
MMSE	28.00 (27.00, 29.00)	29.00 (28.00, 29.75)	0.044^a^
MoCA	22.88 ± 2.64	24.78 ± 2.35	0.003
**Executive function and psychomotor speed**		
TMT-A	76.31 ± 45.42	61.31 ± 35.33	0.145
TMT-B	173.53 ± 85.23	132.59 ± 61.58	0.033
**Mental flexibility**			
VFT	39.75 ± 6.49	42.63 ± 6.30	0.077
**Working memory**			
DST forward	8.84 ± 1.25	9.63 ± 1.66	0.038
DST backward	4.00 (3.00, 4.00)	4.00 (3.00, 4.00)	0.657^a^
**Episodic memory**			
AVLT immediate recall	6.50 ± 1.51	7.18 ± 1.23	0.052
AVLT short-term delayed recall	6.41 ± 3.20	8.13 ± 2.14	0.014
AVLT long-term delayed recall	4.72 ± 3.59	7.00 ± 2.09	0.003
AVLT long-term delayed recognition	10.13 ± 3.60	11.72 ± 2.22	0.038
AVLT total score	27.75 ± 9.56	34.03 ± 6.11	0.003

*p < 0.05 indicates statistically significant. ^a^The Mann-Whitney U-test for non-normally distributed data [median (QR)]. Two sample t-test for normally distributed continuous data (means ± SD).*

### FCD and Functional Connectivity Analyses

**FIGURE 1:**
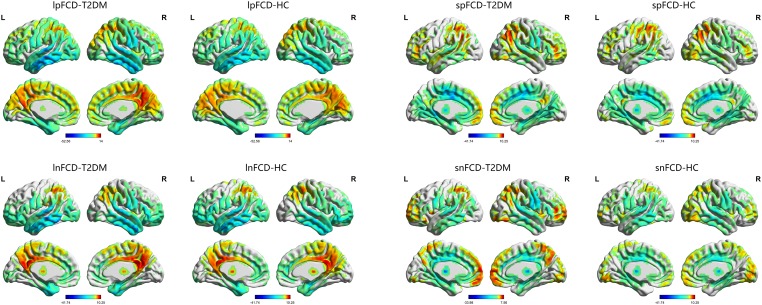
The distribution of FCD in the T2DM group and HC group. Color scale denotes the *t*-value. L, left; R, right.

**FIGURE 2:**
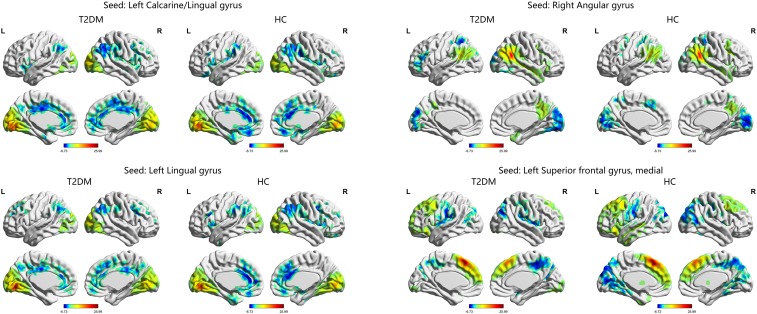
The distribution of functional connectivity of each seed region in the T2DM group and HC group. Color scale denotes the *t*-value. L, left; R, right.

The one-sample *t*-test suggested that both the HC and T2DM groups exhibited higher long-range FCD than the mean brain level primarily in the bilateral posterior cingulate gyri and precuneus, and lower long-range FCD than the mean brain level primarily in the bilateral temporal lobes and frontal lobes; higher short-range FCD primarily in the bilateral calcarine, angular gyri, and frontal lobes, and lower short-range FCD primarily in the bilateral middle cingulate gyri and temporal lobes were also observed (Figure 1). Taking the identified brain areas as seed regions, they showed positive and negative correlations with comprehensive areas of the rest of the brain. That is, the seed regions exhibited functional connectivity throughout the brain (Figure 2).

**FIGURE 3:**
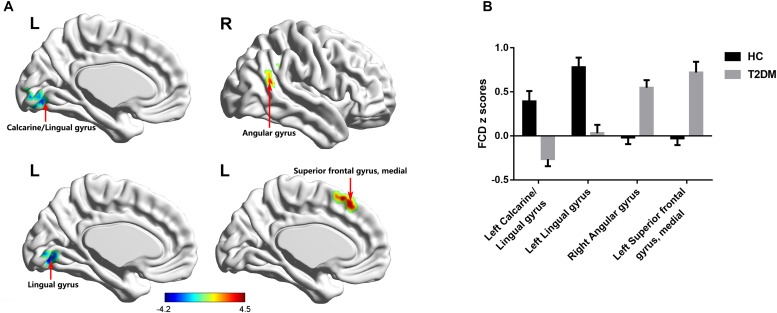
FCD maps of inter-group comparisons. **(A)** The distribution of brain regions with changed FCD in the T2DM group. **(B)** The comparison of FCD z scores between the T2DM group and HC group. Gaussian random-field theory corrected (voxel *p* = 0.01, cluster *p* < 0.05). Color scale denotes the *t*-value. Error bars define the standard error of the mean. L, left; R, right.

Compared with the HC group, T2DM patients showed significantly decreased long-range FCD including lnFCD in the left lingual gyrus and lpFCD in the left calcarine extending to the left lingual gyrus after multiple comparisons correction. In addition, T2DM patients showed increased short-range FCD including snFCD in the medial part of the left superior frontal gyrus and spFCD in the right angular gyrus after multiple comparisons correction (Table 3 and Figure 3). T2DM patients also showed aberrant functional connectivity anchoring in these identified brain regions. However, these results did not survive multiple comparisons correction.

**Table 3:** Brain regions with significant differences in FCD between the T2DM group and HC group.

		Brodmann	Peak MNI coordinates			*T*-values	Voxels	Cluster size
		Area	X	Y	Z			(mm^3^)
lpFCD	Left Calcarine/Lingual gyrus	17/18	-3	-90	0	-4.0559	36	972
lnFCD	Left Lingual gyrus	18	-9	-78	-6	-4.122	41	1107
spFCD	Right Angular gyrus	39/40	54	-51	33	3.8163	44	1188
snFCD	Left Superior frontal gyrus, medial	8	-3	30	51	4.4059	39	1053

*MNI, Montreal Neurological Institute; Gaussian random-field theory corrected (voxel p = 0.01, cluster p < 0.05).*

### Correlation Analyses

**FIGURE 4:**
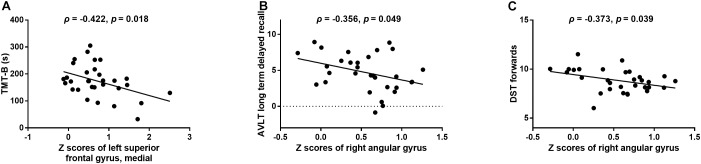
The relationships between changed FCD and aberrant neuropsychological tests in the T2DM group. **(A)** Z scores of FCD in the medial part of the left superior frontal gyrus vs. scores of the TMT-B test. **(B)** Z scores of FCD in the right angular gyrus vs. scores of the AVLT long-term delayed recall. **(C)** Z scores of FCD in the right angular gyrus vs. scores of the DST forward.

In T2DM patients, the z scores of FCD in the medial part of the left superior frontal gyrus were negatively correlated with the time cost of the TMT-B test (*r* = -0.422, *p* = 0.018; Figure 4A). In addition, the z scores of FCD in the right angular gyrus were negatively correlated with the scores of AVLT long-term delayed recall (*r* = -0.356, *p* = 0.049; Figure 4B) and DST forward (*r* = -0.373, *p* = 0.039; Figure 4C). No correlations were observed among the aberrant neuroimaging indicators, blood biometric measurements and other neuropsychological tests.

## Discussion

Previous research proposed that brain networks may negotiate a trade-off between the energy-cost and information propagation efficiency (Bullmore and Sporns, 2012). As the disturbance in glucose metabolism may affect the brain energy homeostasis in T2DM, the present study investigated the disruption in the balance between the long- and short-range FCD. We found that T2DM patients showed decreased long-range FCD in the left calcarine and lingual gyrus and increased short-range FCD in the left superior frontal gyrus and right angular gyrus. These changes were significantly associated with performance on neuropsychological tests in T2DM patients. Our findings may update the insight into T2DM-related brain dysfunction.

The brain regions which exhibited aberrant FCD were reported to be abnormal in previous studies, which suggest that they are susceptible to T2DM. A meta-analysis demonstrated that the superior frontal gyrus and lingual gyrus are robust brain regions with altered resting-state brain activity (Xia et al., 2017). The frontal lobe is involved in executive function and attention that underlies advanced cognition (Xia et al., 2017). Together with occipital regions, the lingual gyrus was considered a brain region involved in vision-related information processing and visual memory encoding (Cui et al., 2016). The lingual gyrus was reported to have a reduced degree of centrality in T2DM patients (Cui et al., 2016), which was similar to that found in the present study. Our previous study also demonstrated aberrant functional connectivity anchoring in the angular gyrus that may serve as a neuroimaging marker for T2DM-related cognitive decline (Liu et al., 2016). With regard to the calcarine, decreased regional homogeneity and amplitude of low frequency fluctuations were observed in T2DM patients, which were associated with cognitive performance (Cui et al., 2014; Peng et al., 2016). In addition to the aforementioned studies, the present study also identified these abnormal brain regions with high centrality due to the similar definition of FCD and degree of centrality (Zuo et al., 2012). According to the computational modeling of neural dynamics, the cerebral cortex with a high degree of centrality plays a pivotal role in global information integration and intermodular communication, which are vulnerable to attack by disease (Bullmore and Sporns, 2012).

The disruption in the balance between long- and short-range FCD may suggest a shift from the costly metabolic connection to an economic connection. Previous studies have reported alterations in long- and short-range functional connectivity strength (Liu et al., 2017), and local and remote brain activity synchronization in T2DM patients (Liu et al., 2016). The present study further suggests that T2DM patients have more short-range connections and fewer long-range connections. There is evidence that the energy-cost of a node increases with the number of connections, and that the energy-cost of a connection increases with length (Sepulcre et al., 2010; Bullmore and Sporns, 2012). Moreover, it was proposed that brain regions with high energy-cost such as hubs and long-distance connections may be sensitive to metabolic distress, and they may reconfigure to achieve variable cognitive demands via the negotiation between connection cost and topological properties of the networks (Kitzbichler et al., 2011; Bullmore and Sporns, 2012). This process of negotiating continues across milliseconds to decades (Bullmore and Sporns, 2012). As with the reduced proportion of long connections in Alzheimer’s disease (Yao et al., 2010), the decreased long-range FCD in T2DM patients may be associated with diminished integrative capacity. We can therefore speculate that the high-cost components in T2DM patients including hubs with high-degree and long-distance connections may be selectively attacked. Furthermore, according to recent findings, the decreased long-range FCD may be prejudicial to the diversity of inputs and outputs in brain areas (Betzel and Bassett, 2018).

Our findings also suggest that the reconfiguration of long- and short-range FCD was associated with the neurocognitive outcomes in T2DM patients. On the one hand, T2DM patients with higher z scores in the right angular gyrus scored lower in the AVLT long-term delayed recall and DST forward tests. This situation is partly similar to patients with autism spectrum disorders whose short-range functional overconnectivity was reported to be positively associated with symptom severity (Keown et al., 2013). The brain regions with preferential short-range connections may be characterized by low energy-cost and high clustering coefficients, but a long path length which have low information propagation efficiency through the network (Sepulcre et al., 2010). On the other hand, the short-range functional overconnectivity of the medial part of the left superior frontal gyrus may play a compensatory role in the better performance of T2DM patients in the TMT-B test. The brain network of children was found to communicate more efficiently due to more short–range interactions and the small world property of the brain network was comparable to that of adults (Fair et al., 2009). A possible explanation for this might be that the reconfigured brain network shares the characteristics of those in children to maintain normal cognitive function. However, the discrepant neurocognitive outcomes of increased short-range FCD require further clarification.

There are several notable limitations in this study. First, this was a cross-sectional study; therefore, the progress of supposed neural compensation cannot be captured and interpretation of the findings should be taken with caution, especially in terms of causality. Second, the functional connections were calculated on the assumption that the time series of distinct brain regions is constant over time. However, this is not ideal for reflecting the dynamic nature of brain activity (Hutchison et al., 2013). Third, the relatively small sample of subjects may restrict the statistical power. Longitudinal studies with a larger sample size to investigate the dynamic connectome may better characterize the diabetic brain and update the neuroimaging evidence of cognitive decline in T2DM patients. Moreover, we conducted global signal regression in the present study to denoise the artifactual fMRI signal, however, it may result in spurious anticorrelations and the global signal fluctuations could reflect true neural variance (Power et al., 2017). Strategies of isolating and removing global artifactual variance while preserving potential global variance may end the controversy on global signal regression in the future.

## Conclusion

In the present study, long- and short-range FCD were used to determine distance information in the brain network of T2DM patients. We found that T2DM patients exhibited increased short-range FCD and decreased long-range FCD which may suggest a trade-off between energy-cost and network efficiency at the expense of losing cognitive function. These findings may improve our understanding and provide potential neuroimaging markers for T2DM brain dysfunction.

## Author Contributions

DL contributed to the experiments, data analysis, and writing of the manuscript. LC contributed to the data analysis and revised the manuscript. SD contributed to data collection. XY contributed to the data analysis. WY contributed to the statistical analysis. YS contributed to the manuscript revision. JZ and JW are the guarantors of this study and had complete access to all data in the study. All authors accept responsibility for the integrity of the data and the accuracy of the data analysis.

## Conflict of Interest Statement

The authors declare that the research was conducted in the absence of any commercial or financial relationships that could be construed as a potential conflict of interest.

## Footnotes

1 http://www.dtu.ox.ac.uk/homacalculator/
